# Serum IL-28A/IFN-λ2 is linked to disease severity of COVID-19

**DOI:** 10.1038/s41598-022-09544-8

**Published:** 2022-03-31

**Authors:** Yosuke Fukuda, Tetsuya Homma, Hideki Inoue, Yuiko Goto, Yoko Sato, Hitoshi Ikeda, Chisato Onitsuka, Hiroki Sato, Kaho Akimoto, Takaya Ebato, Hiromitsu Suganuma, Tomoko Kawahara, Hatsuko Mikuni, Yoshitaka Uchida, Shintaro Suzuki, Akihiko Tanaka, Hironori Sagara

**Affiliations:** Department of Medicine, Division of Respiratory Medicine and Allergology, Showa University School of Medicine, Tokyo, 142-8666 Japan

**Keywords:** Chemokines, Infection, Virology, Translational research

## Abstract

Type III interferons (IFNs) play an important role in respiratory viral infections, including severe acute respiratory syndrome coronavirus 2 (SARS-CoV-2) infection. This study aimed to determine whether the expression of serum type III IFNs predicted disease severity among patients with the coronavirus disease (COVID-19). A retrospective cohort study was conducted of patients admitted to a single hospital between March 21, 2020, and March 31, 2021. Patients were divided into mild to moderate I (MM) and moderate II to severe (MS) groups based on the COVID-19 severity classification developed by the Japanese Ministry of Health, Labor and Welfare. A total of 257 patients were included in the analysis. Human interleukin-28A (IL-28A/IFN-λ2) expression was significantly lower, and interleukin (IL)-6 expression was significantly higher in the MS group than in the MM group (both *p* < 0.001). In addition, IL-28A/IFN-λ2 was statistically significantly inversely correlated with the time from disease onset to negative SARS-CoV-2 PCR results (*p* = 0.049). Multivariable logistic regression analysis showed that IL-28A/IFN-λ2 was an independent predictor of disease severity (*p* = 0.021). The low expression of IL-28A/IFN-λ2 may serve as a serum biomarker that predicts the severity of COVID-19, possibly through the mechanism of delayed viral elimination.

## Introduction

The interferon (IFN) response is a key immune response in respiratory viral infections. Among the IFNs, type III IFNs (IFN-λ) have recently attracted much attention for their antiviral activity. An experimental mouse model of influenza virus infection showed that viral infection resulted in a greater increase in IFN-λ than in IFN-α^1^. Mordstein et al.^2^ reported that mice deficient in both IFN I and IFN III receptors were more susceptible to viral respiratory infections than mice deficient only in IFN I receptors. These previous reports suggest that IFN-λ may play a central role in airway epithelial cells.

It has been reported that IFN-λ inhibits viral replication in severe acute respiratory syndrome coronavirus 2 (SARS-CoV-2) infection but not in severe acute respiratory syndrome coronavirus 1 (SARS-CoV-1) infection^3^. Unlike SARS-CoV-1, SARS-CoV-2 infection induces a large number of IFN-stimulated genes (ISGs), which exert direct antiviral effects^4^. In addition, a previous pilot study showed that serum IFN-λ expression was decreased among patients with severe coronavirus disease (COVID-19) compared to those with mild COVID-19^5^, which is in line with previous reports^6^. Lucas et al.^7^ reported that IFN-λ is one of the cytokines characterizing severe COVID-19 in their cluster analysis of cytokines for moderate-to-severe COVID-19. These findings suggest that IFN-λ may have a valuable function in COVID-19.

However, it is not clear whether the expression of IFNs in COVID-19, especially serum IFNs, which can be measured noninvasively, is a predictor of disease severity. Therefore, in this study, we investigated whether the expression of serum IFN-λ was an independent predictor of disease severity among patients with COVID-19, in combination with previously reported predictors of COVID-19 disease severity.

## Results

Patient characteristics are shown in Table 1. The median age of patients was 55 years, 32% were aged 65 years or older. Patients in the MS group accounted for 29% of the total population.

**Table 1:** Clinical characteristics in total patients.

	*n* = 257
Age, years	55 (37–72)
> 65 years old, *n*	84 (32.7%)
**Sex**	
Male	146 (56.8%)
Female	111 (43.2%)
BMI, kg/m^2^	23.1 (20.4–25.8)
Time from onset to admission, days	5 (3–8)
**Disease severity**^†^, ***n***	
Mild to moderate I	182 (70.8%)
Moderate II to severe	75 (29.2%)
**Treatment regimen,** ***n***	
Systemic corticosteroids	63 (24.5%)
Remdesivir	38 (14.8%)
Favipiravir	49 (19.1%)
Tocilizumab	5 (1.9%)
**Comorbidities,** ***n***	
Hypertension	77 (30.0%)
Diabetes mellitus	27 (10.5%)
Dyslipidemia	46 (17.9%)
Hyperuricemia	9 (3.5%)
Asthma	22 (8.6%)
COPD	13 (5.1%)
Chronic heart failure	33 (12.8%)
Immunodeficiency	14 (5.4%)

The data are presented as median (range) or number (percentage). *BMI* body mass index, *COPD* chronic obstructive pulmonary disease.

^†^Disease severity was based on the classification developed by the Japanese Ministry of Health, Labor and Welfare.

**Figure 1:**
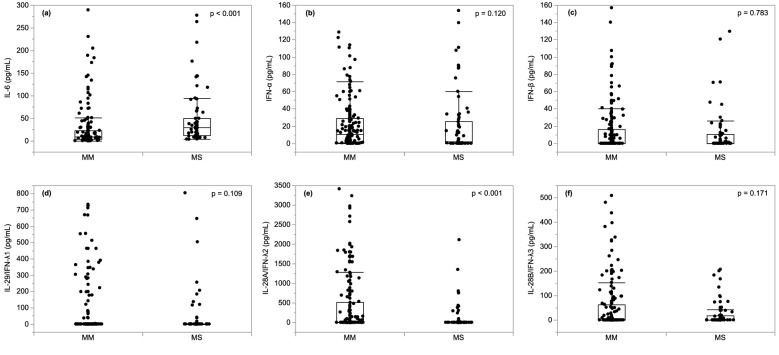
Comparison of serum cytokines levels according to COVID-19 disease severity. The patients were divided into mild to moderate I (MM) (*n* = 182) and moderate II to severe (MS) (*n* = 75) groups based on the COVID-19 severity classification developed by the Japanese Ministry of Health, Labor and Welfare. Data were presented as the median (interquartile range). Compared with the MS group, serum IL-6 level (**a**) was significantly higher (*p* < 0.001), and IL-28A/IFN-λ2 level (**e**) was significantly lower in the MM group (*p* < 0.001). There were no significant differences between the two groups in IFN-α (**b**), IFN-β (**c**), IFN-IL-29/IFN-λ1 (**d**), and IL-28B/IFN-λ3 (**f**). *IL* interleukin, *IFN* interferon, *MM* mild to moderate I, *MS* moderate II to severe.

Figure 1 shows a comparison of serum cytokines between the MM and MS groups in all patients at admission. The expression of IL-6 was significantly higher, and the expression of IL-28A/IFN-λ2 was significantly lower, in the MS group than in the MM group (Fig. 1, both *p* < 0.001). In addition, the MS group had a higher white blood cell count, and higher lactate dehydrogenase, C-reactive protein, ferritin, Kreb von den Lungen-6 levels, and a lower lymphocyte count than the MM group (Supplementary Fig. 1).

To examine factors affecting serum cytokines, we performed a multiple regression analysis of serum IL-6 and IL-28A/IFN-λ2, which were significantly differentially expressed depending on the severity of the disease in this study (Fig. 0.1). Multiple linear regression analysis showed that the presence of immunodeficiency was a factor that significantly affected serum IL-6 and age was a factor that significantly affected IL-28A/IFN-λ2 (Tables 2 and 3).

**Table 2:** Factors associated with serum IL-6 in COVID-19 patients based on multivariate linear regression analysis.

	Estimate	SE	β	t value	*p* value
Age	0.234	0.205	0.095	1.14	0.254
Sex	4.122	3.513	0.081	1.17	0.241
Obesity (BMI > 30 kg/m^2^)	−1.455	6.104	−0.016	−0.24	0.811
Hypertension	1.747	4.319	0.032	0.40	0.686
Dyslipidemia	3.671	4.402	0.056	0.83	0.405
Diabetes mellitus	−10.150	5.872	−0.120	−1.73	0.085
Hyper uric acid	2.618	9.232	0.018	0.28	0.776
Asthma	2.866	5.650	0.032	0.51	0.612
COPD	4.695	8.281	0.038	0.57	0.571
Chronic heart failure	−0.906	7.015	−0.012	−0.17	0.861
Immunodeficiency	−16.266	5.185	−0.150	−2.32	0.021
Smoking history	9.380	6.755	0.108	1.39	0.283

*β* β standardized coefficient, *BMI* body mass index, *COPD* chronic obstructive pulmonary disease, *SE* standardized error.

**Table 3:** Factors associated with serum IL-28A/IFN-λ2 in COVID-19 patients based on multivariate linear regression analysis.

	Estimate	SE	β	t value	*p* value
Age	−8.899	2.669	−0.271	−3.33	0.001
Sex	−27.693	45.495	−0.041	−0.61	0.543
Obesity (BMI > 30 kg/m^2^)	126.226	79.747	−0.108	1.58	0.114
Hypertension	16.606	55.819	−0.023	0.30	0.766
Dyslipidemia	−6.359	57.481	0.060	−0.11	0.912
Diabetes mellitus	−65.037	74.090	0.007	−0.88	0.381
Hyper uric acid	122.592	113.475	−0.069	1.08	0.281
Asthma	98.391	72.934	−0.084	1.35	0.178
COPD	39.811	103.681	−0.025	0.38	0.701
Chronic heart failure	61.856	66.970	−0.063	0.92	0.356
Immunodeficiency	34.196	90.652	−0.023	0.38	0.706
Smoking history	−98.329	86.960	−0.086	−1.13	0.259

*β* β standardized coefficient, *BMI* body mass index, *COPD* chronic obstructive pulmonary disease, *SE* standardized error.

**Figure 2:**
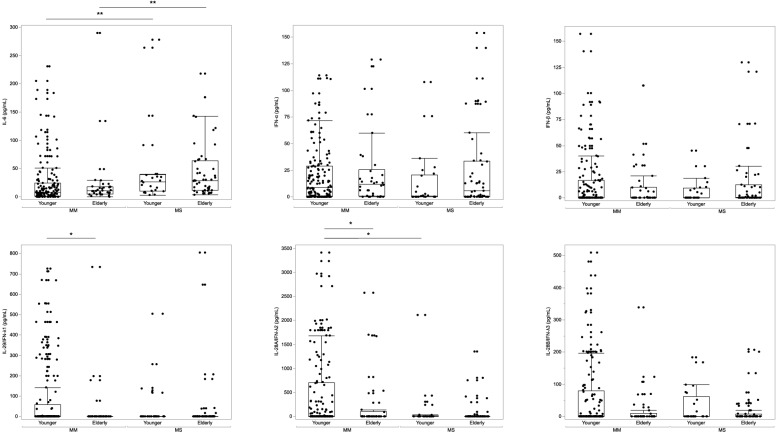
Comparison of serum cytokines levels according to COVID-19 disease severity and age. The patients were divided into mild to moderate I (MM) (*n* = 182) and moderate II to severe (MS) (*n* = 75) groups based on the COVID-19 severity classification developed by the Japanese Ministry of Health, Labor and Welfare. Data were presented as the median (interquartile range). In the younger population, serum IL-6 level (**a**) was significantly higher (*p* < 0.001), and IL-28A/IFN-λ2 level (**e**) was significantly lower in the MS group than in the MM group (*p* < 0.001). In the elderly population, serum IL-6 level (**a**) was significantly higher in the MS group than in the MM group (*p* < 0.001). *IL* interleukin, *IFN* interferon, *MM* mild to moderate I, *MS* moderate II to severe.

Serum cytokine distribution was examined using the kernel smoothing method to clarify the relationship between serum cytokines and age (Supplementary Fig. 2). The results showed that IFN-α and IL-28A/IFN-λ2 were bimodally distributed. We divided the entire patient population into two groups, younger (≤ 65 years) and elderly (> 65 years), and performed cytokine analysis (Fig. 2). In the younger population, IL-6 was significantly higher (*p* = 0.001), and IL-28A/IFN-λ2 was significantly lower (*p* = 0.022) in the MS group. In the elderly population, only IL-6 was significantly higher in the MS group than in the MM group (Fig. 2, *p* < 0.001).

**Figure 3:**
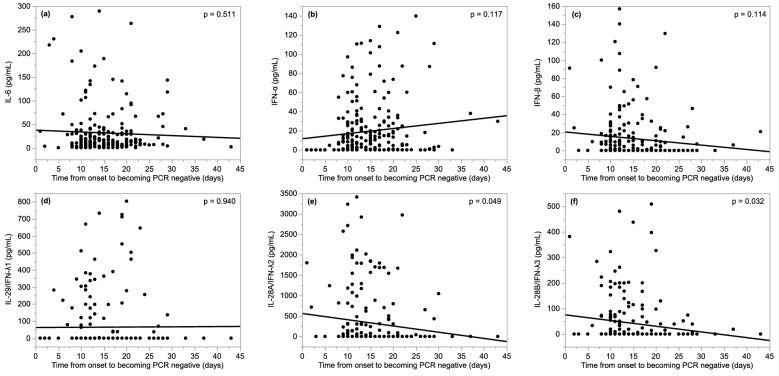
Correlation between serum cytokines and time from disease onset to becoming negative for SARS-CoV-2 on polymerase chain reaction testing. The expression of serum IL-28A/IFN-λ2 and IL-28B/IFN-λ3 were significantly inversely correlated with time from disease onset to becoming negative PCR for SARS-CoV-2 (*p* = 0.049; r = −0.13, *p* = 0.032; r = −0.14, respectively). *IL* interleukin, *IFN* interferon, *PCR* polymerase chain reaction, *SARS-CoV-2* severe acute respiratory syndrome coronavirus 2.

Figure 3 shows the correlation between serum cytokines and the time from disease onset to becoming PCR negative. IL-28A/IFN-λ2 and IFN-28B/IFN-λ3 were inversely correlated with the time from disease onset to becoming PCR negative (*p* = 0.049, *p* = 0.032, respectively). Although we examined cytokines and time from onset to becoming negative PCR in each MM and MS group, there was no statistically significant correlation (Supplementary Fig. 3).

A multiple regression analysis of markers of disease severity using known risk factors^8–11^, IL-6, and IL-28A/IFN-λ2 revealed that IL-28A/IFN-λ2 was an independent predictor of severe disease (*p* = 0.021, Table 4). Age older than 65 years, sex, and obesity were also independent predictors of severe disease.

**Table 4:** Risk factors associated with COVID-19 severity based on multivariable logistic regression analysis.

	OR	95% CI	*p* value
Age	1.090	1.060–1.120	< 0.001
Sex	0.341	0.156–0.749	0.007
Obesity (BMI > 30 kg/m^2^)	4.770	1.360–16.70	0.014
Hypertension	1.760	0.813–3.800	0.152
Dyslipidemia	0.416	0.172–1.010	0.052
Diabetes mellitus	2.420	0.836–7.020	0.103
IL-6	0.998	0.995–1.001	0.224
IL–28A/IFN–λ2	0.998	0.997–0.999	0.021

*BMI* body mass index, *CI* confidence interval, *IFN* interferon, *IL* interleukin, *OR* odds ratio.

## Discussion

This study revealed that the expression of IL-28A/IFN-λ2 in serum was low in patients with severe COVID-19. This result was consistent with the results of a previous study^5^. Furthermore, we showed that serum IL-28A/IFN-λ2 was an independent factor for COVID-19 severity and that serum IL-28A/IFN-λ2 was inversely correlated with the time to becoming PCR negative.

The importance of IFN-λ has been demonstrated in viral infections, such as influenza. The addition of IL-29/IFN-λ1 to alveolar type II epithelial cells induces antiviral genes such as myxovirus resistance protein 1,2'-5'-oligoadenylate synthetase 1, and ISG56^12^. Experiments using a mouse model of influenza A virus infection of the upper and lower respiratory tracts showed that IFN-λ exerted antiviral effects for a more extended period than IFN-α^13^. Moreover, it was reported that in the serum of patients infected with Puumala hantavirus, IFN-λ expression was reduced by about 9% in the acute phase compared with the recovery phase^14^. In vitro studies comparing SARS-CoV-1 and SARS-CoV-2 showed that although viral replication was higher in SARS-CoV-2 infection than in SARS-CoV-1 infection, the expression of IFN-λ1,2,3 in lung tissue was not different between SARS-CoV-2 and controls^15^. The expression of serum IFN-λ was maintained in children regardless of the severity of COVID-19^16^, suggesting that the disease severity is relatively lower in children than in adults. These findings suggest that IFN-λ may be important in preventing severe disease. In the present study, multivariable analysis revealed that serum IL-28A/IFN-λ2 was an independent predictor of disease severity in COVID-19, after controlling for severity factors found in several previous studies. Collectively, serum IFN-λ, especially IL-28A/IFN-λ2, may be a biomarker of disease severity in COVID-19.

The present study also revealed that IL-29/IFN-λ1 and IL-28A/IFN-λ2 were weakly, but certainly statistically inversely correlated with the time until becoming SARS-CoV-2 PCR became negative. Experiments using human alveolar epithelium have found that IFN-λ is induced by SARS-CoV-2 infection and persistently inhibits SARS-CoV-2 replication for 7 days^17^. IFN-β and IFN-λ1 have been shown to inhibit SARS-CoV-2 replication in a dose-dependent manner^18^. In contrast to type I IFNs, expressed in the liver, spleen, brain, and other parenchymal organs, type III IFNs are expressed predominantly in the airway epithelium and gastrointestinal epithelium^2,19^. In these epithelia, plasmacytoid dendritic cells control viral infections by releasing IFN-λ. It has been found that patients with COVID-19 experience a prolonged reduction in dendritic cell function, which is more pronounced in severely ill patients^20^. In older patients, activation of factors that control signal transduction, including PI3K signaling, is reduced, and it is possible that virus excretion may be delayed due to the reduced function of plasmacytoid dendritic cells^21–23^. Another study showed that prostaglandin D2 receptor 1 promotes viral elimination by increasing the production of IL-28A^24^. This study did not reveal the mechanism by which IL-28A/IFN-λ2 and IL-28B/IFN-λ3 were inversely correlated with the time to PCR negativity in COVID-19, so further investigation is needed.

One of the main strengths of this study is that it demonstrated that IL-28A/IFN-λ2, a type III IFN, was an independent predictor of COVID-19 severity. Identifying factors that predict disease severity is important for defining treatment targets. Although it is unclear how type III interferon acts on COVID-19, one possible explanation is that type III IFNS including IL-28A/IFN-λ2 may contribute to the prevention of severe disease by promoting viral shedding.

However, there were some limitations to the study. First, this was a single-center study conducted over a relatively short period of time. The number of patients is also not sufficient to analyze all confounding factors reported so far. Second, we did not analyze changes in the serum cytokine concentrations over time. It is important to determine whether serum IFN recovers after treatment. A study comparing serum cytokines in COVID-19 and seasonal influenza found that serum levels of IFN-λ1 and IFN-α remained low for at least 3 weeks in patients with COVID-19^25^. Third, it is unclear why patients with severe disease had a greater reduction in serum IL-28A/IFN-λ2 than other types of type III IFN-λ. Similar to our results, it has been report that IL-28A/IFN-λ2 plays an essential role in COVID-19 pathogenesis^26^. Some previous reports have shown that IL-29/IFN-λ1 and IL-28B/IFN-λ3 are markers of COVID-19 disease activity^25,27^. Fourth, we could not demonstrate the downregulation of type III IFNs in severe COVID-19 in the elderly (Supplementary Fig. 1). The reasons could be due to small number of participants or the total amount of expression of type III IFNs are generally less among elderly population.

In summary, this study showed that serum IL-28A/IFN-λ2 is linked to viral excretion of SARS-CoV-2, and its expression level may be associated with the severity of COVID-19. As serum IL-28A/IFN-λ2 is decreased in severe COVID-19, IL-28A/IFN-λ2 supplementation may be an appropriate treatment for COVID-19. A randomized placebo-controlled trial showed that a single subcutaneous injection of peginterferon lambda safely and significantly shortened viral shedding duration compared to placebo^28^. Although, various drugs that control the cytokine storm, such as corticosteroids, anti-IL-6 antibodies, and Janus kinase inhibitors, are thought to be effective against severe COVID-19, some patients with severe disease do not recover with these drugs alone or in combination. Therefore, further studies focusing on serum IFNs are needed to develop novel treatments for COVID-19.

## Methods

This observational study was conducted from March 21, 2020 to March 31, 2021 at Showa University Hospital in Tokyo, Japan. We included 257 patients over 16 years of age who had confirmed the diagnosis of COVID-19 based on a positive polymerase chain reaction (PCR) test using a nasal swab. Based on the COVID-19 severity classification developed by the Ministry of Health, Labor and Welfare^29^, we separated the patients into mild–moderate I (MM) and moderate II–severe (MS) groups.

We reviewed the medical records and collected the following data: age, sex, body mass index, comorbidities, and blood test results on the first day of hospitalization. All data were fully anonymized before they were accessed. Additionally, we measured the following serum cytokines and chemokines: interleukin (IL)-6, interferon (IFN)-α, IFN-β, IL-29/IFN-λ1, IL-28A/IFN-λ2, and IL-28B/IFN-λ3. The serum concentrations of cytokines and chemokines were measured using enzyme-linked immunosorbent assay (ELISA) kits (R&D Systems, Minneapolis, USA). The ELISAs were performed according to the manufacturer's instructions. A negative PCR for SARS-CoV-2 was defined as two consecutive Ct values above 30.

Statistical analyses were performed using EZR (Saitama Medical Center, Jichi Medical University, Saitama, Japan) and JMP version 15 (SAS Institute, Minato-ku, Tokyo, Japan). All data were presented as the median (interquartile range) or the frequency (percentage), as appropriate. The validity of the normal distribution was assessed using the Shapiro–Wilk test. Fisher's exact test and the Mann–Whitney U test were used to assess the statistical significance of differences between categorical variables and continuous variables, respectively. Correlations between pairs of continuous variables were analyzed using Pearson's correlation coefficient. We conducted a multivariate logistic regression analysis to evaluate risk factors for COVID-19 severity. Independent variables included potential risk factors based on previous reports^8–11^ and serum IL-28A/IFN-λ2. In addition, we estimated the variance inflation factors for the variables and confirmed that there was no multicollinearity to prove the validity of these parameters. To analyze factors affecting serum cytokines, specifically IL-28A/IFN-λ2, multiple linear regression analysis was performed combining age, gender, obesity status, and history of obesity. ANOVA test was used to confirm the significance of the analysis. We excluded the points that exceeded 1.5 times the upper and lower limits of the interquartile range as outliers. *P* values less than 0.05 were considered statistically significant.

The study was conducted in accordance with the Declaration of Helsinki and was approved by the Showa University Ethics Committee (approval number: 3300). Informed consent was obtained from all patients on the day of admission.

## Supplementary Information

Supplementary Information 1.

Supplementary Information 2.

Supplementary Information 3.

Supplementary Information 4.

Supplementary Information 5.

Supplementary Information 6.

## Data Availability

The datasets generated during and/or analyzed during the current study are available from the corresponding author on reasonable request.
