# Supplementary material for: Serum IL-28A/IFN-λ2 is linked to disease severity of COVID-19
Source: Sci Rep. 2022 Mar 31;12:5458. doi: 10.1038/s41598-022-09544-8 (PMC8969403; doi:10.1038/s41598-022-09544-8)
Supplement: Supplementary file 1 — Supplementary Information 1. [file 41598_2022_9544_MOESM1_ESM.docx]

**Title**

Serum IL-28A/IFN-λ2 is linked to SARS-CoV-2 excretion and severity of COVID-19

**Authors**

Yosuke Fukuda^1^, Tetsuya Homma^1*^, Hideki Inoue^1^, Yuiko Goto^1^, Yoko Sato^1^, Hitoshi Ikeda^1^, Chisato Onitsuka^1^, Hiroki Sato^1^, Kaho Akimoto^1^, Takaya Ebato^1^, Hiromitsu Suganuma^1^, Tomoko Kawahara^1^, Hatsuko Mikuni^1^, Yoshitaka Uchida^1^, Shintaro Suzuki^1^, Akihiko Tanaka^1^, Hironori Sagara^1^

**Supplementary Figure 1.** Comparison of serum biomarkers according to COVID-19 disease severity. The patients were divided into mild to moderate I (MM) (n = 182), and moderate II to severe (MS) (n = 75) groups based on the COVID-19 severity classification developed by the Japanese Ministry of Health, Labor and Welfare. Data were presented as the median (interquartile range). Compared with the MS group, the white blood cell count (a) was significantly higher (p < 0.001); the lymphocyte count (b) was significantly lower (p < 0.001); and the lactate dehydrogenase (LDH) (c), C-reactive protein (CRP) (d), ferritin (e), and Kreb von den Lungen-6 (KL-6) (f) levels were significantly higher (all p < 0.001), in the MM group. *CRP* C-reactive protein, *KL-6* Kreb von den Lungen-6, *LDH* lactate dehydrogenase, *MM* mild to moderate I, *MS* moderate II to severe.
