# Supplementary figures and images for: Serum IL-28A/IFN-λ2 is linked to disease severity of COVID-19
Source: Sci Rep. 2022 Mar 31;12:5458. doi: 10.1038/s41598-022-09544-8 (PMC8969403; doi:10.1038/s41598-022-09544-8)

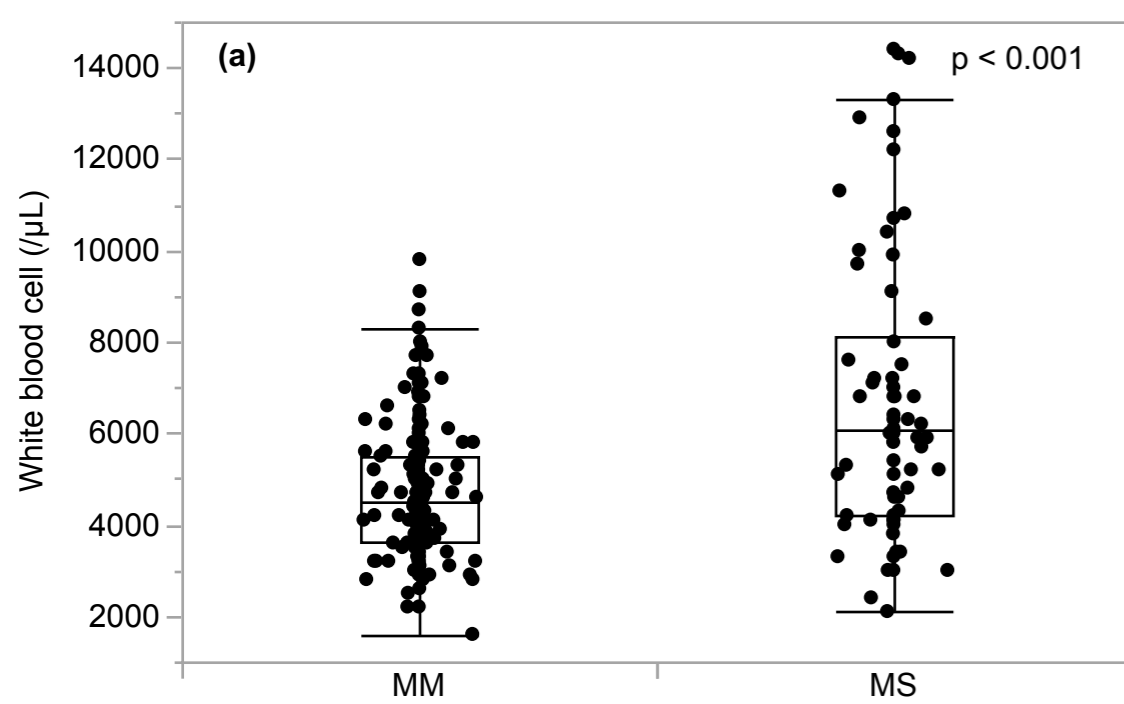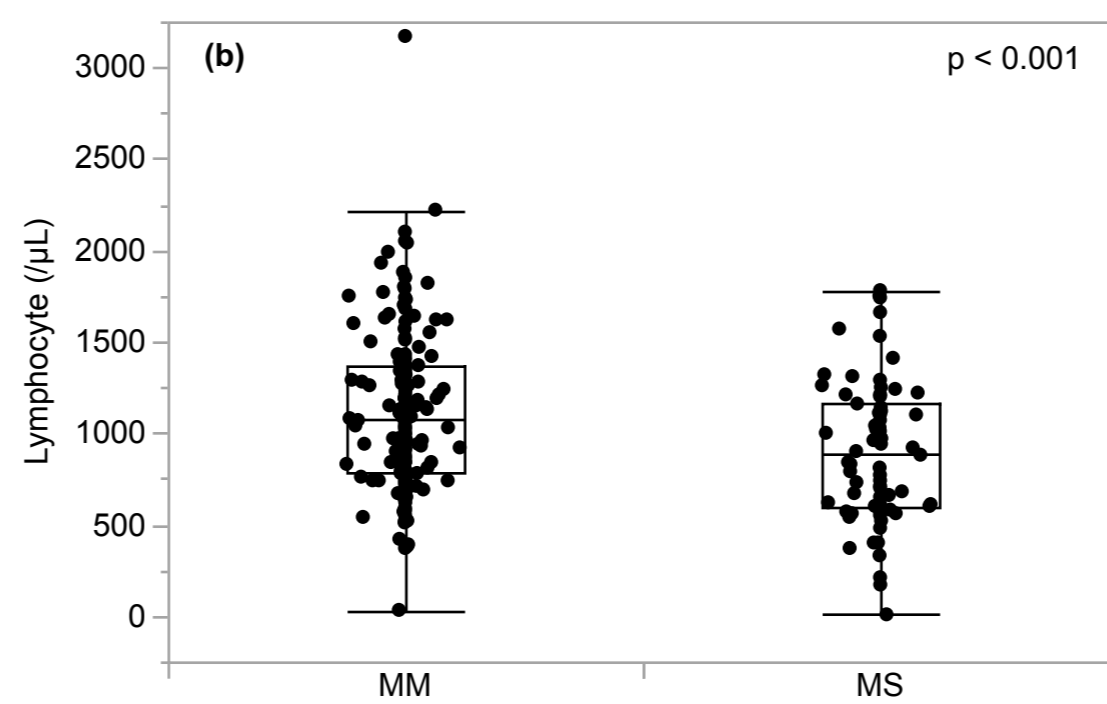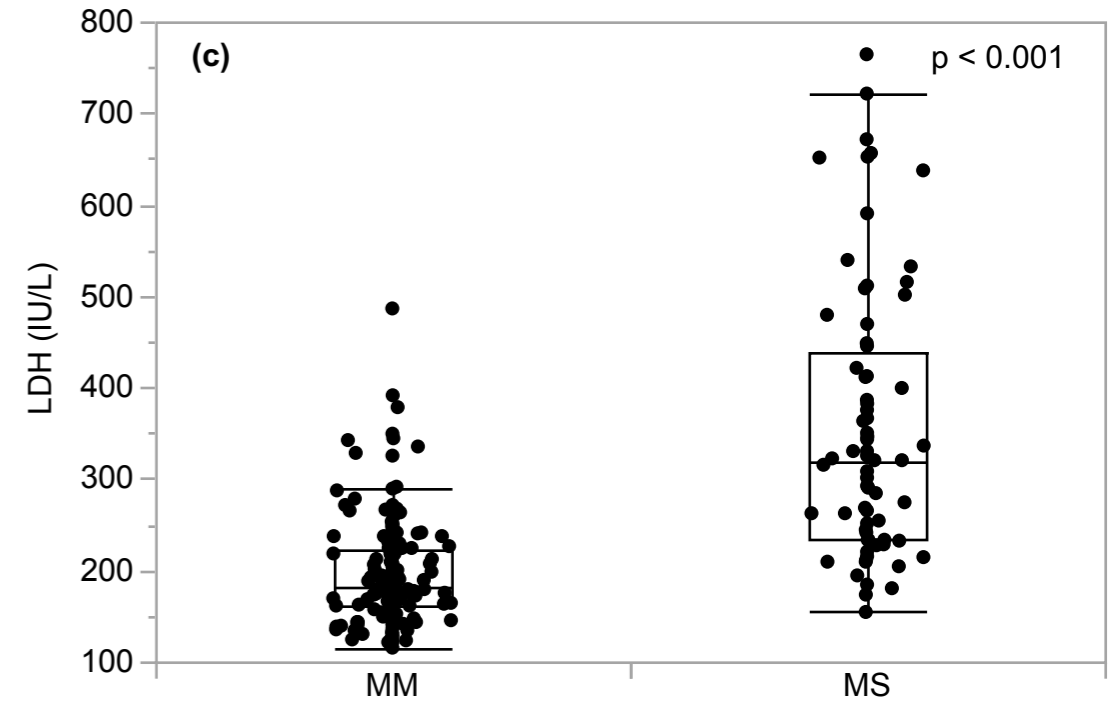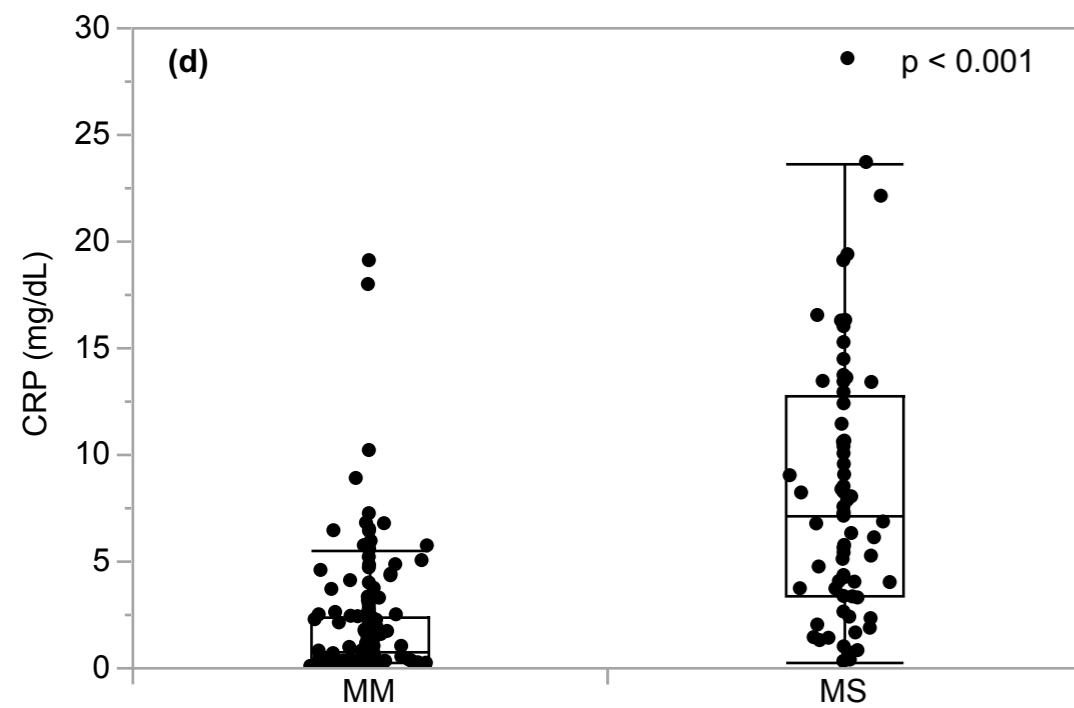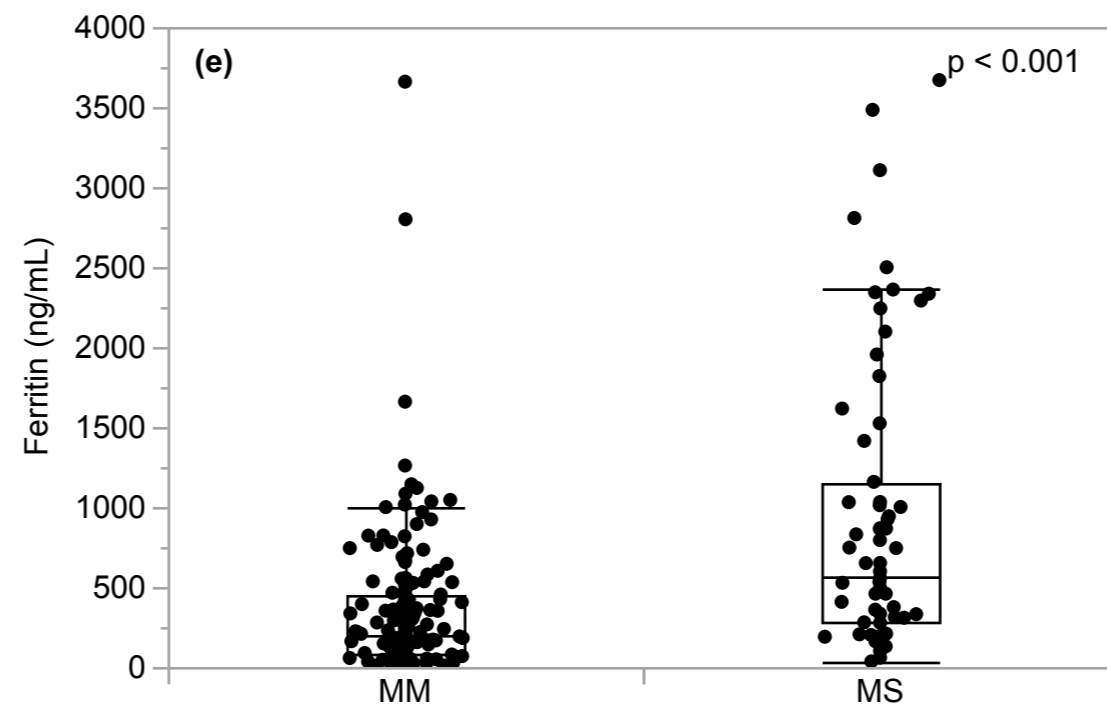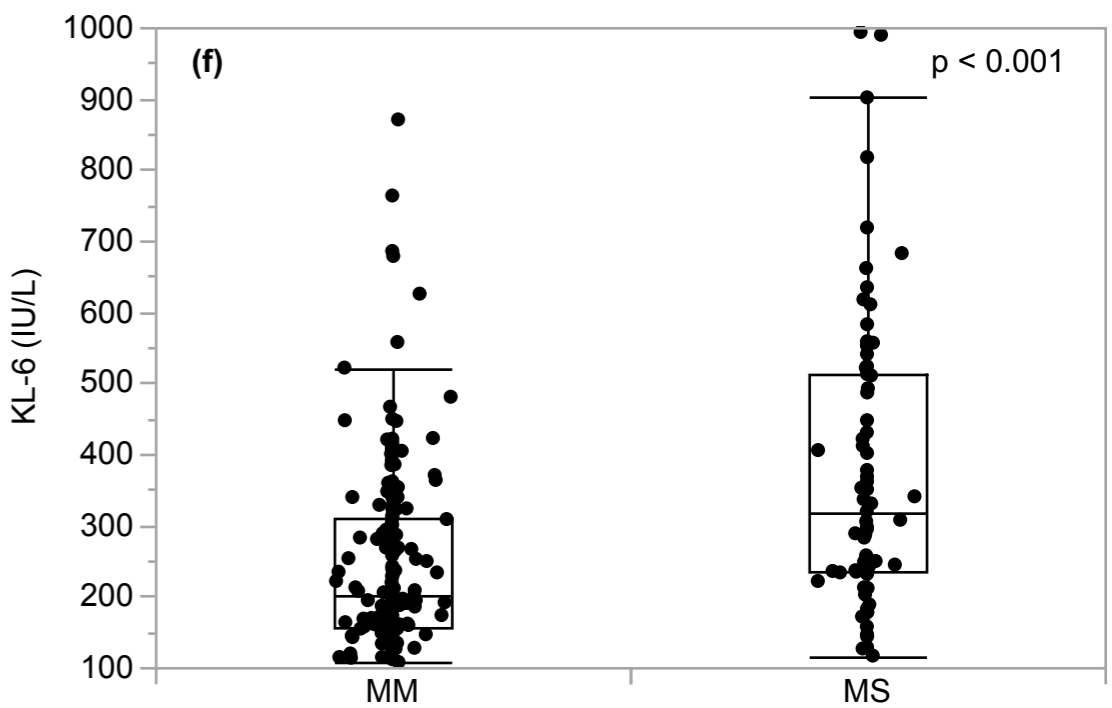

Supplement: Supplementary file 2 — Supplementary Information 2. [file 41598_2022_9544_MOESM2_ESM.pdf]

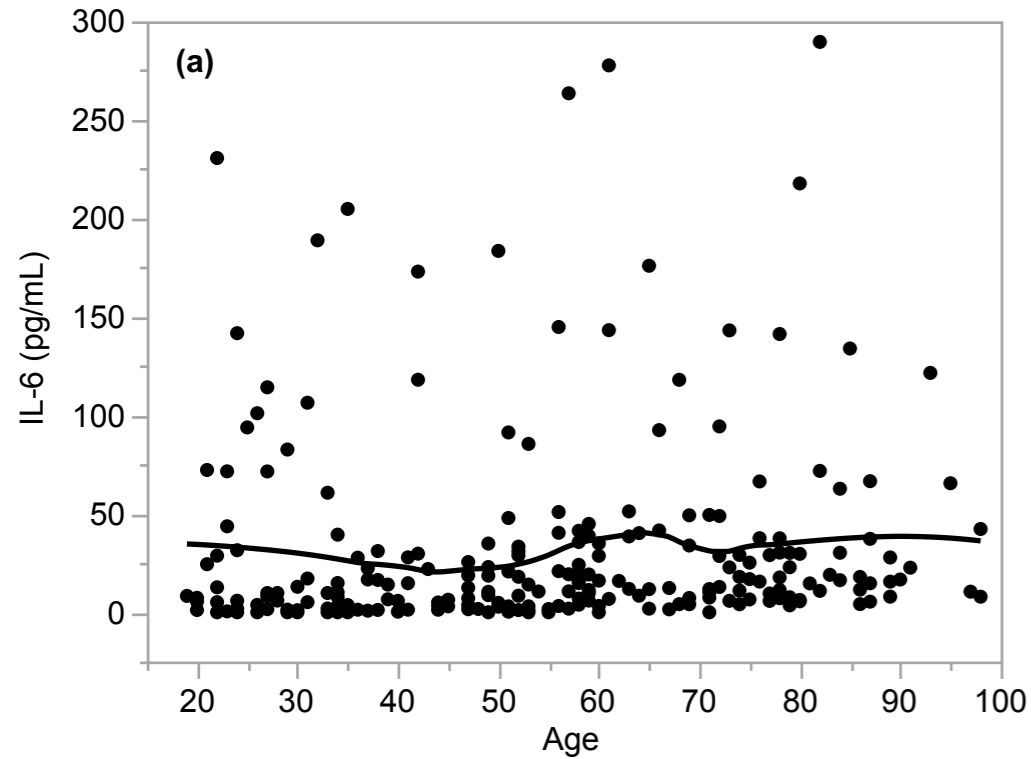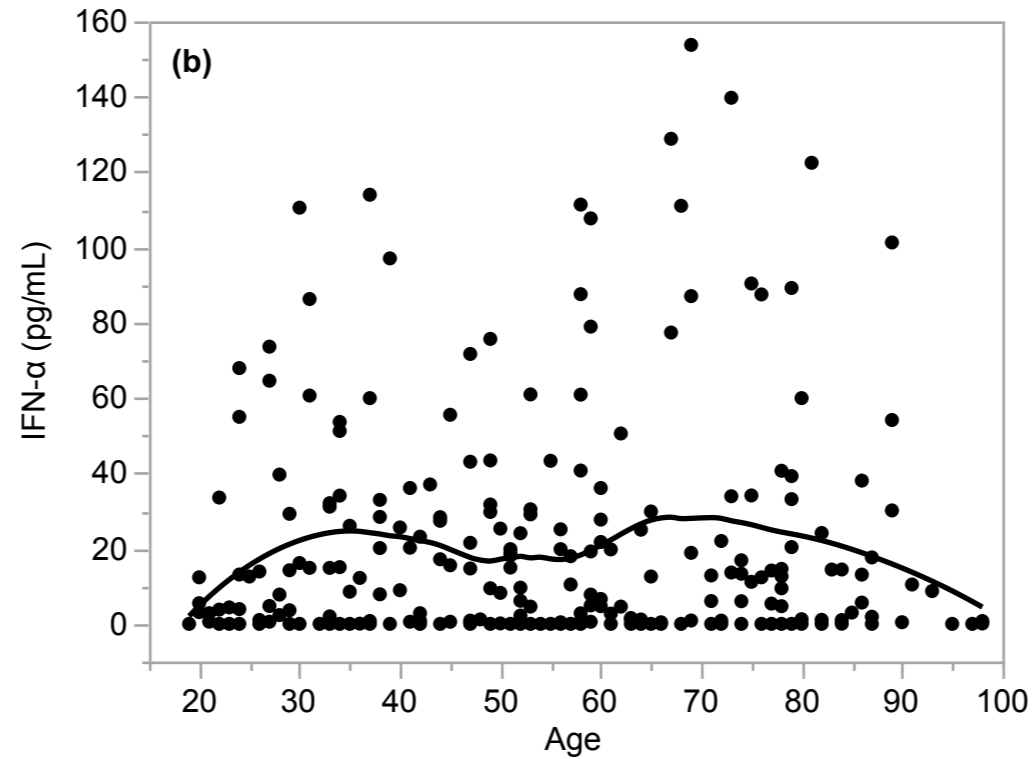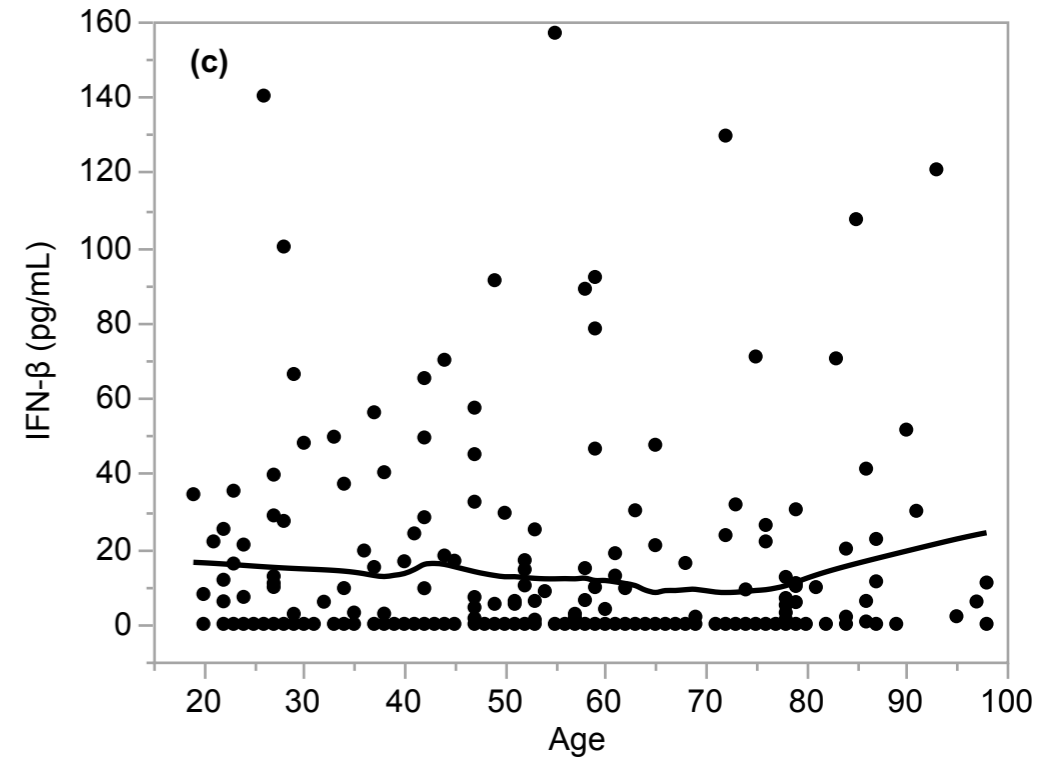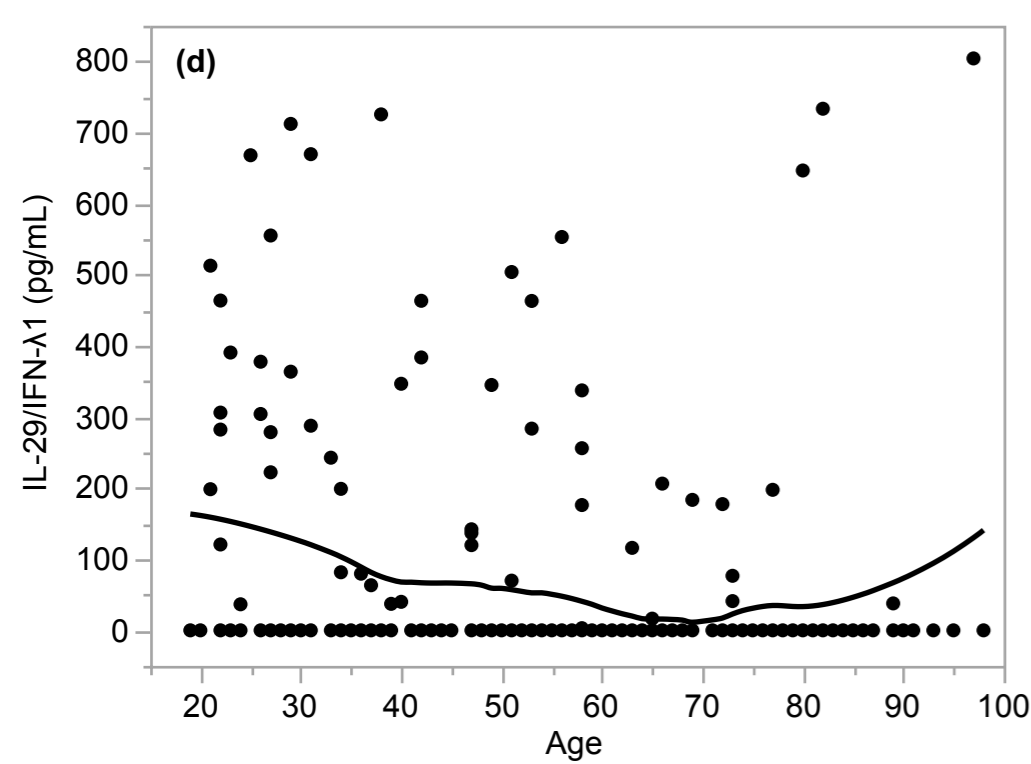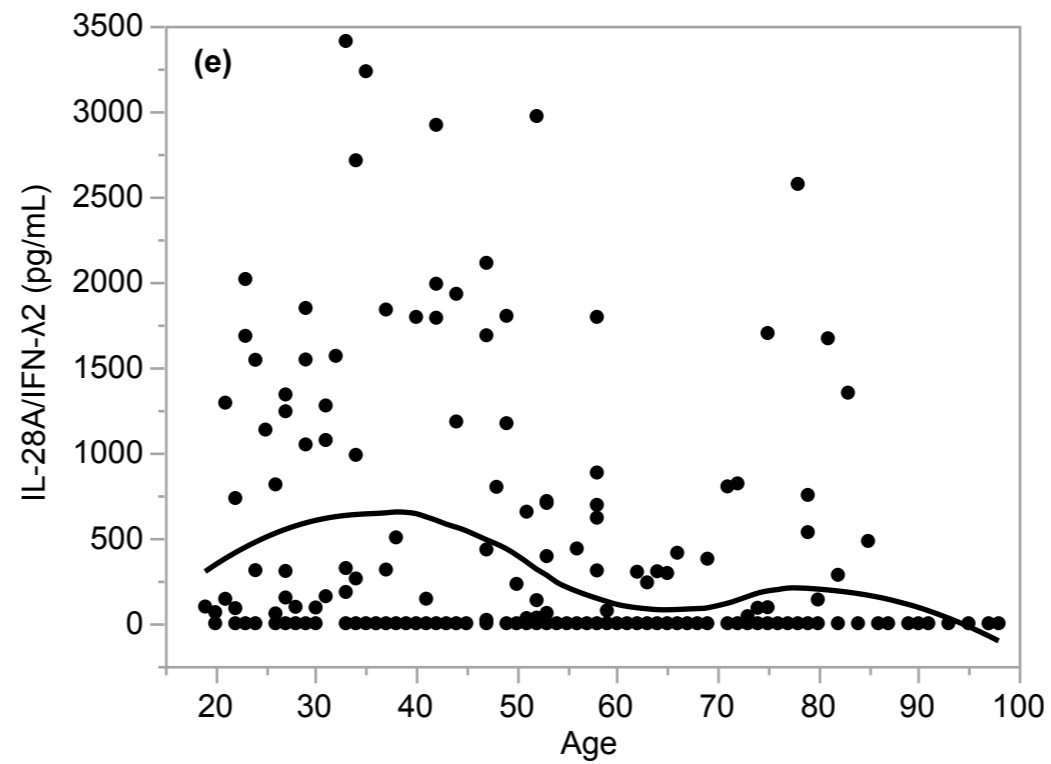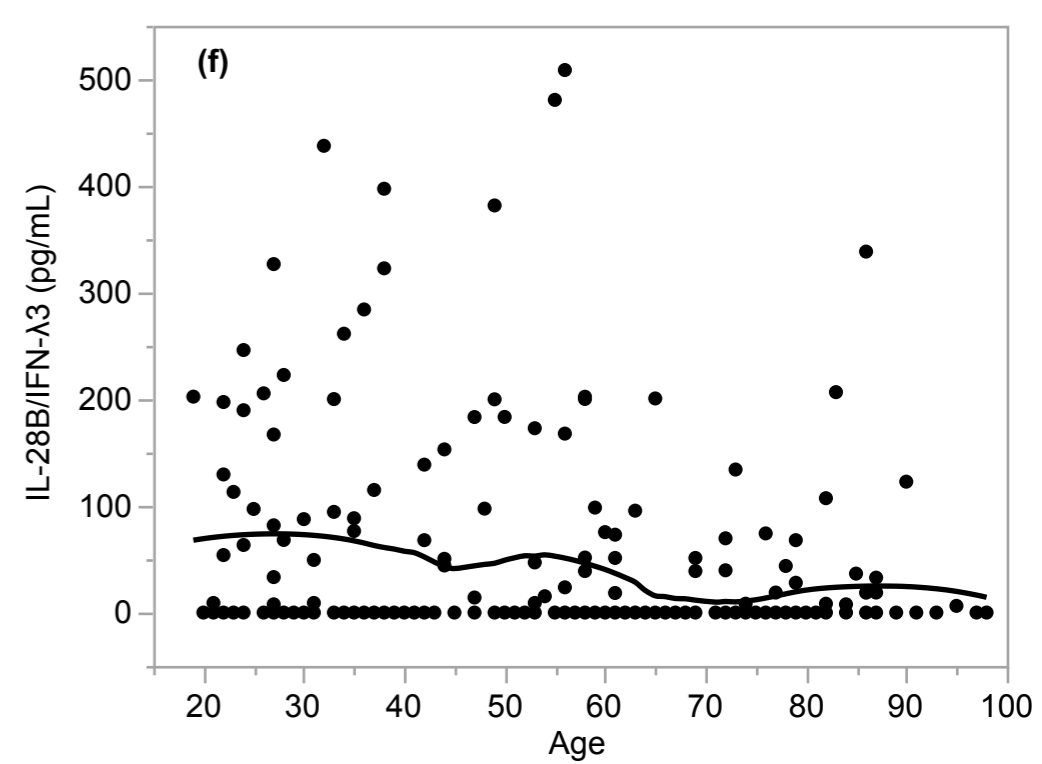

Supplement: Supplementary file 4 — Supplementary Information 4. [file 41598_2022_9544_MOESM4_ESM.pdf]

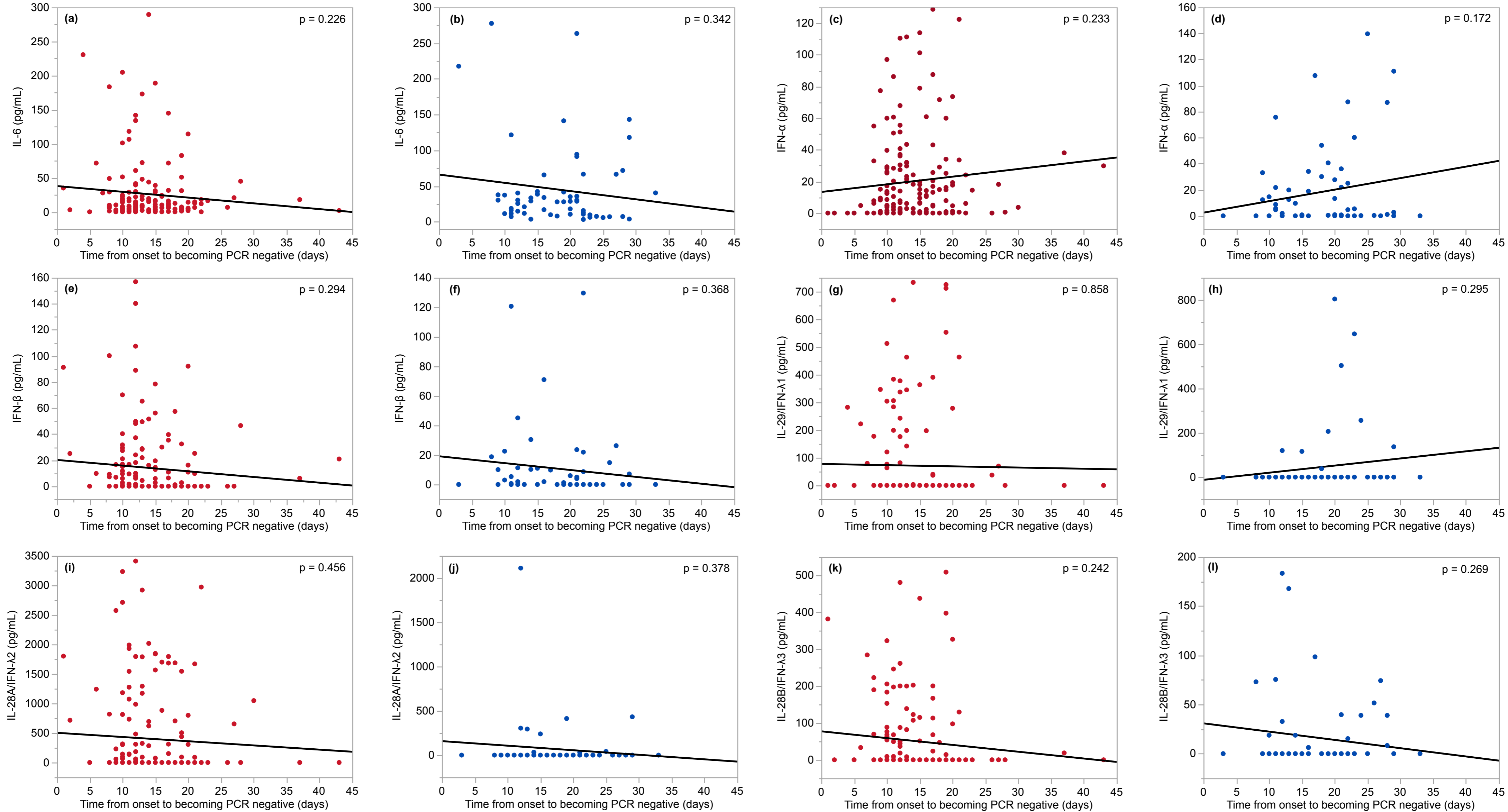

Supplement: Supplementary file 6 — Supplementary Information 6. [file 41598_2022_9544_MOESM6_ESM.pdf]
