# Supplementary material for: Serum IL-28A/IFN-λ2 is linked to disease severity of COVID-19
Source: Sci Rep. 2022 Mar 31;12:5458. doi: 10.1038/s41598-022-09544-8 (PMC8969403; doi:10.1038/s41598-022-09544-8)
Supplement: Supplementary file 5 — Supplementary Information 5. [file 41598_2022_9544_MOESM5_ESM.docx]

**Title**

Serum IL-28A/IFN-λ2 is linked to SARS-CoV-2 excretion and severity of COVID-19

**Authors**

Yosuke Fukuda^1^, Tetsuya Homma^1*^, Hideki Inoue^1^, Yuiko Goto^1^, Yoko Sato^1^, Hitoshi Ikeda^1^, Chisato Onitsuka^1^, Hiroki Sato^1^, Kaho Akimoto^1^, Takaya Ebato^1^, Hiromitsu Suganuma^1^, Tomoko Kawahara^1^, Hatsuko Mikuni^1^, Yoshitaka Uchida^1^, Shintaro Suzuki^1^, Akihiko Tanaka^1^, Hironori Sagara^1^

**Supplementary Figure 3.** Correlation between serum cytokines and time from disease onset to becoming negative for SARS-CoV-2 on polymerase chain reaction testing by severity**.** The red dots are for the MM group, and the blue dots are for the MS group. There was no statistically significant correlation. *IL* interleukin, *IFN* interferon, *PCR* polymerase chain reaction, *SARS-CoV-2* severe acute respiratory syndrome coronavirus 2.
